# Supplementary material for: A self-sustaining endocytic-based loop promotes breast cancer plasticity leading to aggressiveness and pro-metastatic behavior
Source: Nat Commun. 2020 Jun 15;11:3020. doi: 10.1038/s41467-020-16836-y (PMC7296024; doi:10.1038/s41467-020-16836-y)
Supplement: Supplementary file 1 — Supplementary Information [file 41467_2020_16836_MOESM1_ESM.pdf]

SUPPLEMENTARY INFORMATION

A self-sustaining endocytic-based loop promotes breast cancer plasticity leading to aggressiveness and pro-metastatic behavior

Irene Schiano Lomoriello, *et al.*

This PDF file contains:

- Supplementary Figures and Legends 1-10
- Supplementary Tables and Legends 1-6

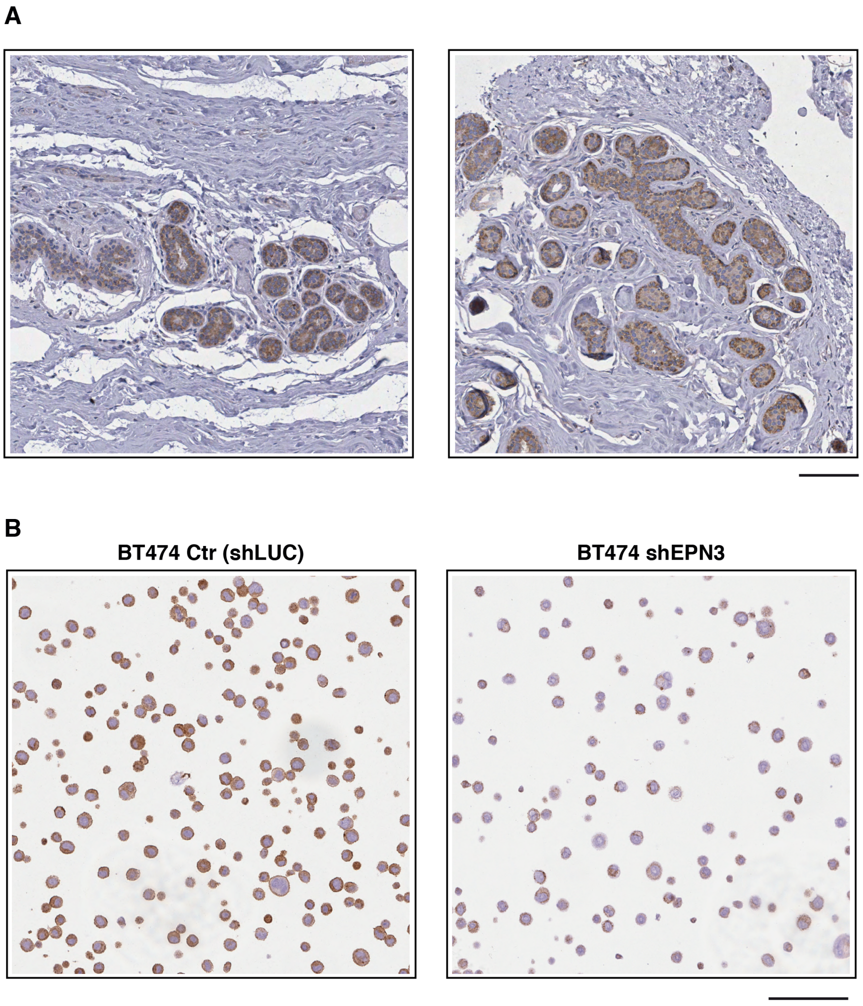

Supplementary Figure 1. Expression of EPN3 in the normal human mammary gland and EPN3 antibody specificity. (**A**) EPN3 stained (IHC score ≤1.0) the luminal and/or the basal layer of the normal mammary gland with comparatively higher expression in scattered myoepithelial cells. Bar, 100 μm. (**B**) BT474 cells were subjected to stable silencing of EPN3 (shEPN3) or mock-silenced with an irrelevant construct (shLUC). After fixation and paraffin embedding, to mimic the conditions of IHC in whole tissue FFPE blocks, blocks were cut (3 μm slices) and analyzed by IHC. Representative images were taken at 20X magnification. Bar, 100 μm. Source data are provided as a Source Data file.

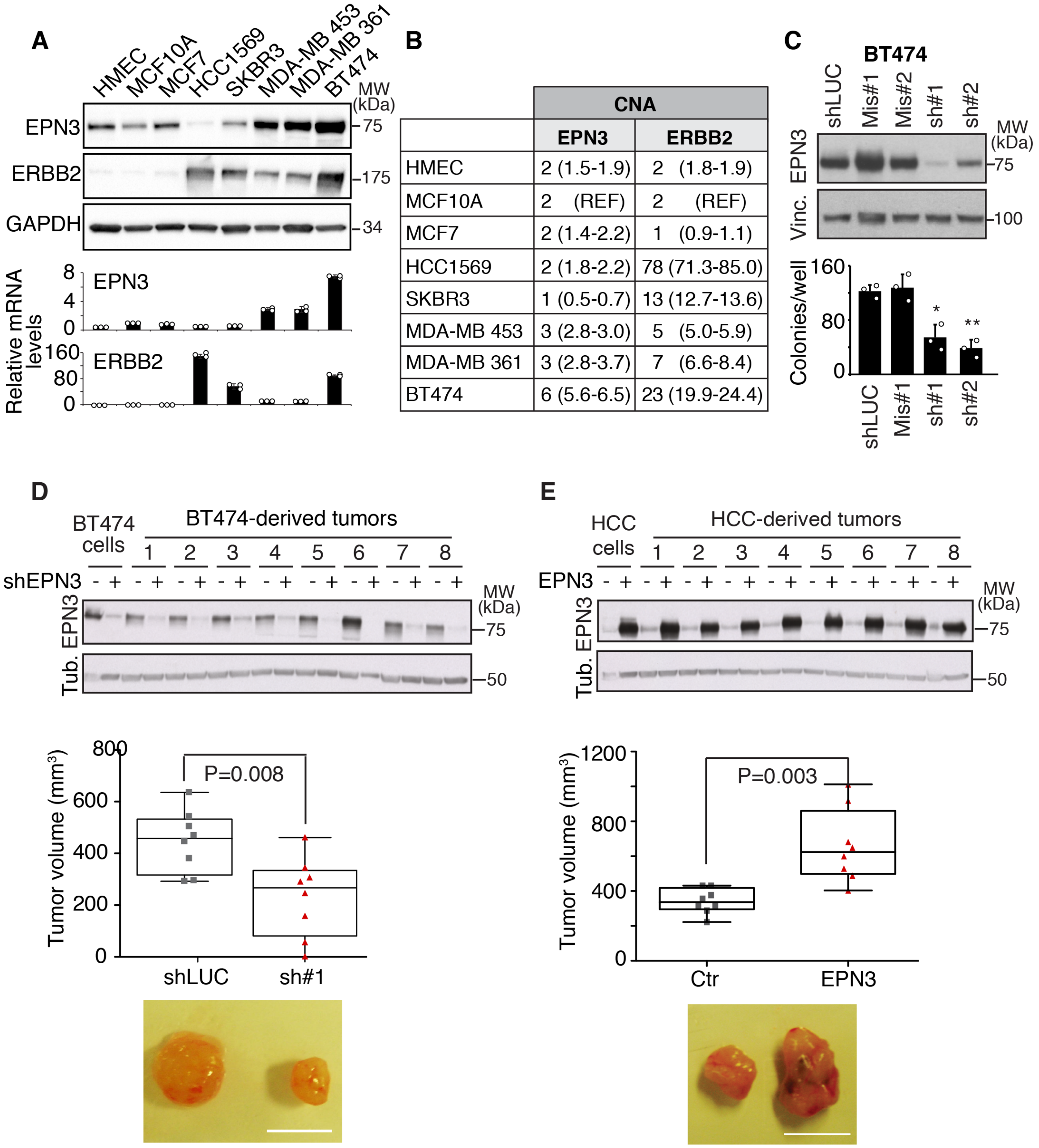

	CNA	
	EPN3	ERBB2
HMEC	2 (1.5-1.9)	2 (1.8-1.9)
MCF10A	2 (REF)	2 (REF)
MCF7	2 (1.4-2.2)	1 (0.9-1.1)
HCC1569	2 (1.8-2.2)	78 (71.3-85.0)
SKBR3	1 (0.5-0.7)	13 (12.7-13.6)
MDA-MB 453	3 (2.8-3.0)	5 (5.0-5.9)
MDA-MB 361	3 (2.8-3.7)	7 (6.6-8.4)
BT474	6 (5.6-6.5)	23 (19.9-24.4)

Supplementary Figure 2. EPN3 expression levels influence the tumorigenic potential of BC cell lines. (**A**) Analysis of EPN3 and ERBB2 expression in human breast epithelial and BC cell lines. Top, IB analysis in the indicated cell lines (HMEC and MCF10A are "normal" lines, all others are tumor lines). GAPDH, loading control. MW markers are shown on the right. Bottom, RT-qPCR analysis. Data were normalized on *GAPDH* and results are reported as relative mRNA

expression compared to the MCF10A cells, mean ± 95% confidence interval (CI) of three independent experiments, each performed in technical triplicates. (**B**) Summary table of *EPN3* and *ERBB2* copy number analysis (CNA) performed by RT-qPCR in the panel of breast cell lines shown in (**A**). MCF10A were used as a calibrator sample (2n genome for chromosome 17). Copy number values were rounded to the nearest whole integer. Minimum and maximum values of three independent PCR experiments are indicated in parentheses. (**C**) Anchorage-independent growth of BT474 cells [displaying EPN3 overexpression and gene amplification, see panels (**A**) and (**B**)] depends on EPN3 overexpression. BT474 cells were infected with lentiviral vectors (pSICOR) expressing shRNAs targeting EPN3 (sh#1, sh#2). Lentiviral vectors encoding shRNA targeting luciferase (shLUC) or mismatch shRNAs (Mis#1, Mis#2) were used as negative controls. Top, efficiency of stable silencing in BT474 was assessed by IB. Vinculin, loading control. MW markers are shown on the right. Bottom, the anchorage-independent growth of BT474-infected cells was assessed using the soft agar assay. Bar graphs report mean colony number per well ± S.D. of an experiment performed in triplicate, and are representative of three independent experiments. P-values (Student's t-test two-tailed) *vs.* shLUC. (**D**) EPN3 ablation affects *in vivo* tumor growth of BT474 cells displaying amplification and overexpression of EPN3 (panels **A** and **B**). Tumors were generated by injecting BT474 cells, either silenced for EPN3 (KD, sh#1) or mock-silenced (shLUC), into opposite inguinal mammary fat pads of a female NOD/SCID IL2 gamma-chain null (NSG) mice. Tumors were grown for 13 weeks before being explanted. Top, the expression of EPN3 in the developed tumors was assessed by IB with the anti-EPN3 antibody. Tubulin (Tub), loading control. MW markers are shown on the right. Middle, distribution of tumor volume of BT474 shLUC and sh#1 is reported in a box-plot. Results are representative of 2 experiments (N=8). Bottom, representative images of tumor

outgrowths are shown. Bar, 11 mm. P, P-value, Student's t-test two-tailed. (**E**) EPN3 overexpression increases the tumorigenic potential of BC HCC1569 (HCC) cells harboring "physiological" EPN3 levels (panels **A** and **B**). HCC1569 cells were infected with a lentiviral vector expressing EPN3 (pLVX-EPN3, EPN3) or empty vector as control (pLVX, Ctr). Tumors were generated by injecting EPN3-overexpressing HCC1569 (EPN3) or control vector-infected (Ctr) cells, into opposite inguinal mammary fat pads of the same female NSG mice. Tumors were grown for 43 days before being explanted. Top, the expression of EPN3 in parental cells and in the developed tumors was assessed by IB. Tubulin (Tub), loading control. MW markers are shown on the right. Middle, the distribution of tumor volume of HCC1569 pLVX-EPN3 (EPN3) and pLVX (Ctr) is reported in a box-plot. Results are representative of 2 experiments (N=8). Bottom, representative images of tumor outgrowths are shown. Bar, 11 mm. P-value, Student's t-test two-tailed. Source data are provided as a Source Data file.

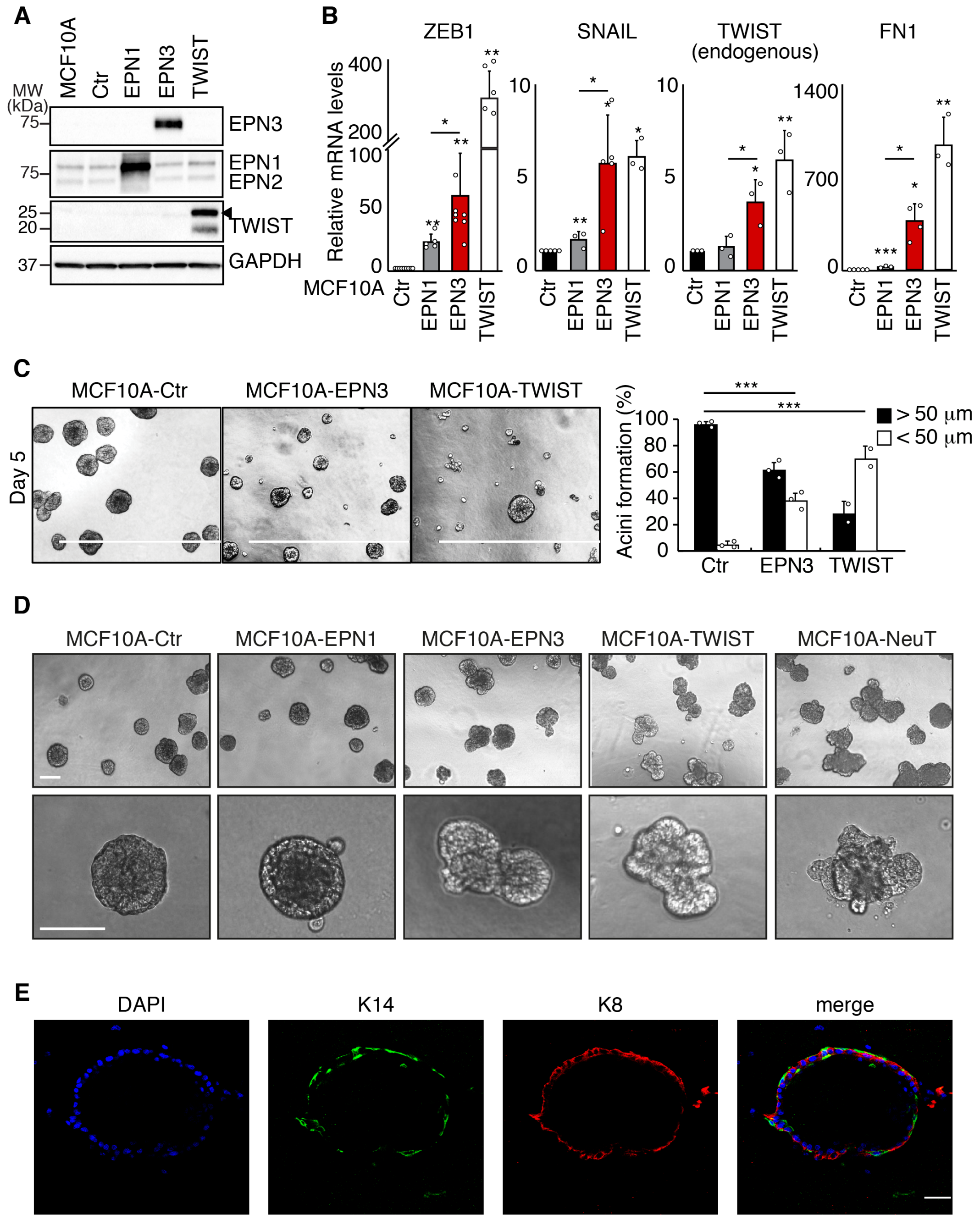

Supplementary Figure 3. EPN3 overexpression in breast cells induces partial EMT and aberrant acini formation in Matrigel. (**A**) MCF10A cells were infected with pBABE retroviral vectors expressing Flag-EPN3 (EPN3), Flag-EPN1 (EPN1) or TWIST (see also Figures 2A-C). Cells infected with empty vector were used as controls (Ctr). GAPDH, loading control. MW markers are shown on the left. Arrowhead indicates the band corresponding to overexpressed

TWIST full-length. The lower band in the IB most likely corresponds to a degradation product, whose abundance varies among experiments (see, for instance, Figure 8G). (**B**) RT-qPCR analysis of mRNA expression of ZEB1, SNAIL, TWIST (endogenous levels) and FN1 in MCF10A-Ctr, -EPN1, -EPN3 and -TWIST cells. Results are reported as relative mRNA expression compared to MCF10A-Ctr, mean ± S.D. of at least three independent experiments, each performed in technical triplicates. P value, Student's t-test two tailed. (**C**) Left, representative bright-field images of *acini* grown in Matrigel for 5 days (bar, 1000 μm). Right, quantification of the percentage of *acini* formation after 5 days. Briefly, organoids from pictures acquired at day 5 of independent experiments (n=3 for Ctr and EPN3; n=2 for TWIST) were counted using ImageJ software (v1.52, NIH); their diameters were measured to identify organoids larger or smaller than 50 μm. A two-sided Fisher's exact t test for 2x2 contingency table was used to analyze statistical significance. (**D**) MCF10A cells overexpressing the indicated constructs were grown in Matrigel for 21 days to allow for the formation of *acini*-like structures. MCF10A expressing the NeuT oncogene were used as positive control for altered morphogenesis. Two representative bright-field microscopy images of *acini* at low (top) or high (bottom) magnification are shown. Bars, 200 μm. (**E**) Bilayered organoids obtained from mammary gland of EPN3-KI mice not treated *in vitro* with Cre were subjected to IF with Ab against keratin14 (K14, myoepithelial marker) and keratin8 (K8, luminal marker). Blue, DAPI. Bar, 45 μm. Source data are provided as a Source Data file.

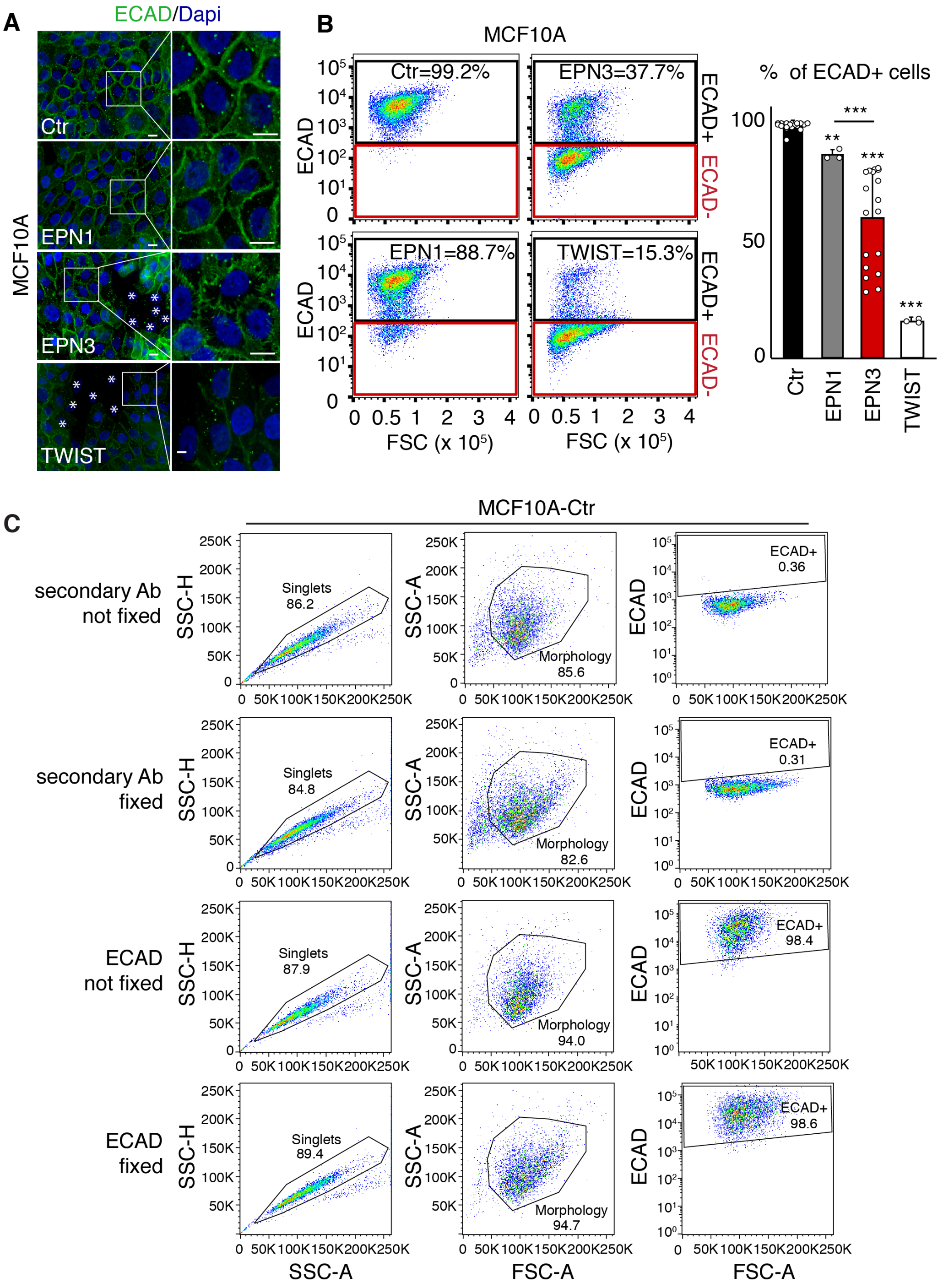

Supplementary Figure 4. Additional control for ECAD IF- and FACS-based experiments. (**A**) IF staining of ECAD with an antibody directed against its intracellular domain (green) in the indicated cells at confluency. Blue, DAPI. Asterisks mark cells lacking ECAD staining. Bar, 10 µm. MCF10A-Ctr and -EPN3 pictures are the same shown in Figure 4A. (**B**) Representative

FACS analysis of MCF10A-Ctr, -EPN1, -EPN3 and -TWIST cells stained *in vivo* for ECAD. Black and red gates represent ECAD-positive and -negative cells, respectively, and have been set according to the unstained control samples. FSC, forward scatter. The percentage of ECAD-positive cells in one out of at least three biological replicates is indicated. Quantification of the percentage of ECAD-positive cells is shown on the right, mean ± S**.**D**.** (n=18 for Ctr and EPN3 samples, the same of Figure 4B; n=3 for EPN1 and Twist). P value, Student's t-test two-tailed. (**C**) Gating strategy for FACS analysis and comparison of ECAD staining prior or after fixation. MCF10A-Ctr cells were subjected to EGF-starvation for 3 hrs, then stained with anti-ECAD antibody (Abcam, 2 µg ml^{-1}) for 1 h at 4°C, followed by incubation with secondary antibody (Alexa-488, 5 µg ml^{-1}) for 30 min at 4°C. Cells were then washed twice with PBS and 0.25% trypsin was added for 15-20 min at 37°C. Cells were recovered in medium, washed once with PBS and fixed in 2% formaldehyde or left untreated (not fixed). Subsequently, cells were centrifuged 335 xg for 5 min and re-suspended in PBS with EDTA 2 mM analyzed with the FACS Celesta (BD). Samples stained only with secondary antibody were used as negative control. Analysis was performed using the FlowJo Software (v10.4.2, LLC). Briefly, cell doublets and clumps were excluded through the SSC-A (Side Scatter Area) over the height peak parameter (SSC-H), panels on the left. Cells with correct morphology were then selected through the FSC-A (Forward Scatter Area) over SSC-A, panels in the middle. Finally, ECAD mean fluorescence intensity of the population over FSC-A was used to compare the different samples, panels on the right. The same gating strategy based on exluding doubltes and clumps through SSC-A *vs* SSC-H and on correct morphology of cells through FSC-A *vs* SSC-A was used for all FACS and FACS-sorting experiments. Source data are provided as a Source Data file.

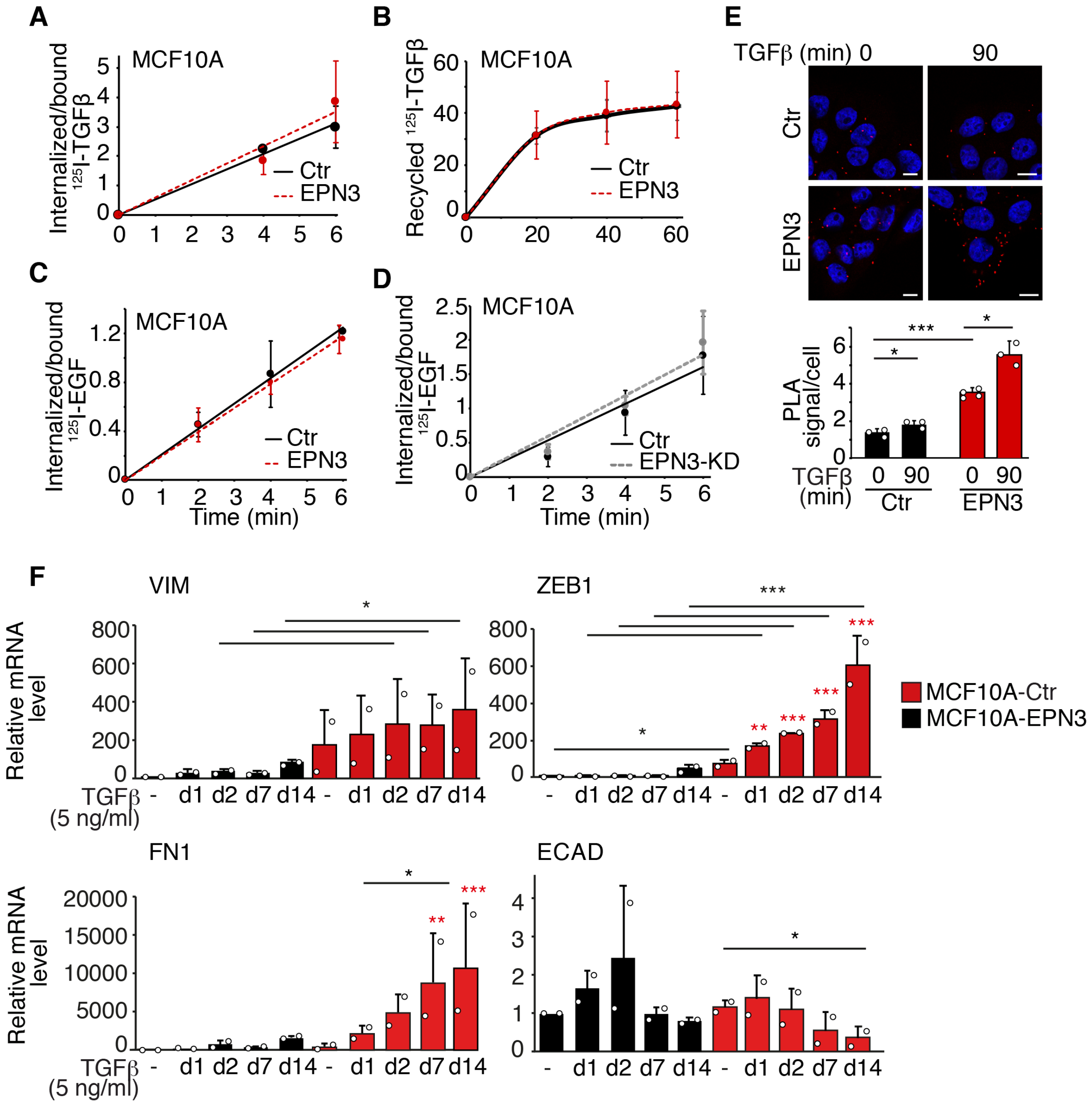

Supplementary Figure 5. Specific role for EPN3 in ECAD internalization. (**A**) ^{125}I-TGFβ internalization rate measured in MCF10A-Ctr or -EPN3 cells. Data are the mean of internalized/bound ^{125}I-TGFβ ± S.D. of two independent experiments, each performed in technical triplicates. (**B**) ^{125}I-TGFβ recycling assay in MCF10A-Ctr or -EPN3 cells. Intact ^{125}I-TGFβ released in the cell medium (recycled ^{125}I-TGFβ) was measured at the indicated time

points (see Methods). Data are the mean of recycled ^{125}I-TGFβ ± S.D. of two independent experiments, each performed in technical triplicates. (**C**) ^{125}I-EGF internalization rate measured in MCF10A-Ctr or -EPN3 cells. Data are the mean of internalized/bound ^{125}EGF ± S.D. of three independent experiments, each performed in technical triplicates. (**D**) ^{125}I-EGF internalization rate measured in MCF10A-Ctr or EPN3-KD cells. Data are the mean of internalized/bound ^{125}EGF ± S.D. of two independent experiments, each performed in technical triplicates. In (**A-D**), no significant differences were scored by Student's t-test two-tailed. (**E**) MCF10A-Ctr and MCF10A-EPN3 cells were stimulated with TGFβ for 90 min or cultured without stimuli (0), followed by analysis of the ECAD-EPN3 interaction by *in situ* Proximity Ligation Assay (PLA, Duolink). Top, representative images are shown. Red, Duolink signal. Blue, Dapi. Bar, 10 µm. Bottom, quantification of the mean PLA signal/cell ± S.D. (at least three field of view for each sample) was performed by ImageJ software (v1.52, NIH). P value, Student's t-test two-tailed. (**F**) MCF10A-Ctr and -EPN3 cells were stimulated for the indicated time points with TGFβ1 (5 ng ml^{-1}), and analyzed for VIM, ZEB1, FN1 and ECAD mRNA levels by RT-qPCR. Results are reported as relative mRNA expression compared to MCF10A-Ctr (not stimulated), mean ± S.D. of two independent experiments, each performed in technical triplicates. P value, Pair Student's t-test two-tailed. Black asterisks indicate statistical significance between each corresponding time point of MCF10A-EPN3 *vs.* -Ctr cells, as indicated by the bar; red asterisks indicate statistical significance between stimulated samples *vs.* not stimulated MCF10A-EPN3 cells. Source data are provided as a Source Data file.

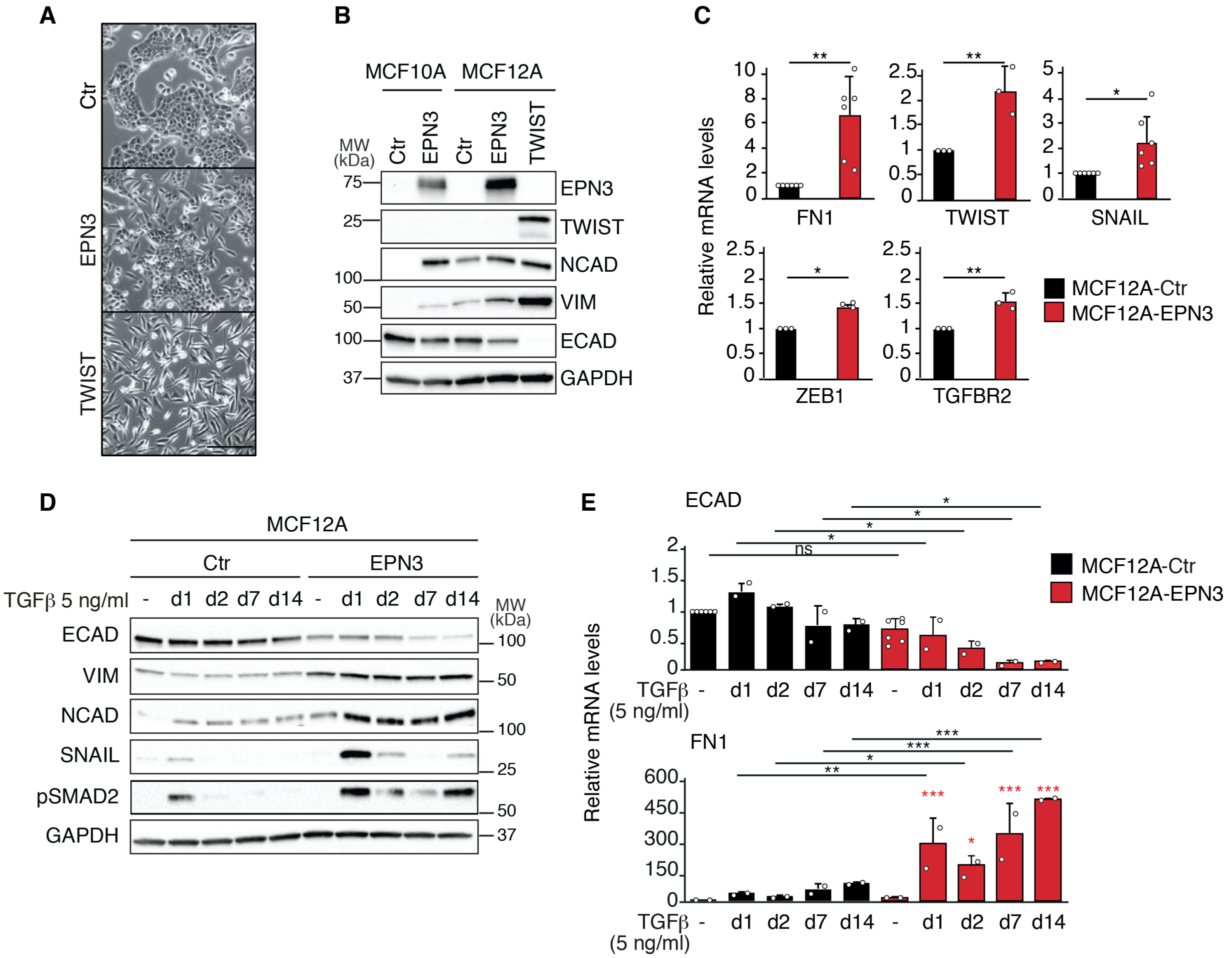

Supplementary Figure 6. MCF12A cells overexpressing EPN3 recapitulate the partial EMT phenotype of MCF10A. (A) MCF12A cells were infected with pBABE empty vector (Ctr), or pBABE-EPN3 or pBABE-TWIST. Representative phase contrast microscopy pictures of MCF12A cells are shown. Bar, 250 μm. (**B**) Lysates of MCF12A cells described in (**A**) were IB with the indicated Ab (MCF10A-Ctr or -EPN3 cells were used as controls). GAPDH, loading control. MW markers are shown on the left. (**C**) RT-qPCR of the indicated EMT markers in MCF12A-Ctr or MCF12A-EPN3 cells. Results are reported as relative fold change compared to MCF12A-Ctr, mean ± S.D. of independent experiments (n=6 for FN1 and SNAIL; n=3 for TWIST, ZEB1 and TGFβR2). P value, Student's t test, two tailed. (**D**) MCF12A-Ctr and -EPN3 cells were stimulated with 5 ng ml^{-1} of TGFβ for the indicated time points (d, days), followed by

IB with the indicated Ab. GAPDH, loading control. MW markers are shown on the right. (**E**) MCF12A-Ctr and -EPN3 cells treated as in (**D**) were analyzed for ECAD and FN1 mRNA levels by RT-qPCR. Data were normalized on the expression of the house keeping gene *18S*, and results are reported as relative mRNA expression compared to MCF12A-Ctr (not stimulated), mean ± S.D. of at least two independent experiments, each performed in technical triplicates. P value, Each Pair Student's t-test two-tailed. Black asterisks indicate statistical significance between each corresponding time point of MCF12A-EPN3 *vs.* -Ctr, as indicated by the bar; red asterisks indicate statistical significance between stimulated MCF12A-EPN3 samples *vs.* the not stimulated one.

Note that EPN3 overexpression in MCF12A resulted in phenotypes closely resembling those obtained in MCF10A cells, including: i) the acquisition of a mesenchymal elongated phenotype (panel A); ii) the upregulation of the mesenchymal markers NCAD and VIM, at the protein level (panel B), and of FN1, SNAIL, TWIST, ZEB1, and TGFβR2, at the mRNA level (panel C). ECAD protein levels were only slightly decreased, while its mRNA levels were not significantly downregulated, in agreement with what we have observed in MCF10A cells. Importantly, TWIST overexpression caused a much more advanced EMT as compared to EPN3, confirming that EPN3 phenotype could configure with a partial EMT also in this setting. Stimulation with TGFβ further increased the EPN3-induced partial EMT phenotype, causing the upregulation of NCAD, SNAIL and pSMAD2 at the protein level (panel D), the upregulation of FN1 mRNA levels (panel E, bottom) and the downregulation of ECAD at both protein (panel D) and mRNA level (this latter occurring in EPN3 overexpressing, but not in control cells, panel E, top). Source data are provided as a Source Data file.

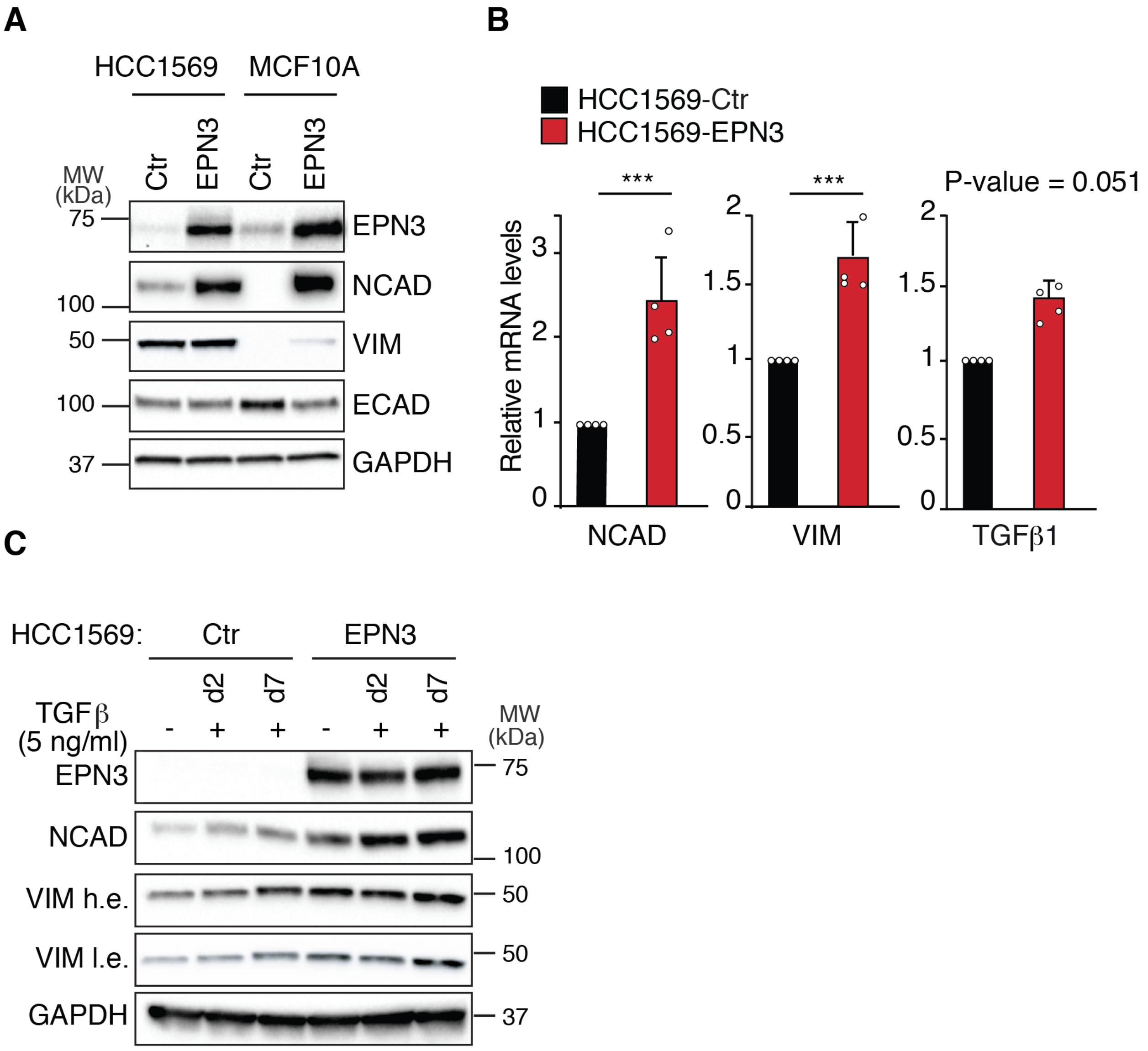

Supplementary Figure 7. Characterization of EPN3 ectopic overexpression in breast cancer HCC1569 cells. (**A**) HCC1569 cells were infected with pLVX empty vector (Ctr) or pLVX vector expressing EPN3 and analyzed by IB analysis for the expression of EPN3, ERBB2, and EMT markers in comparison to MCF10A-Ctr or -EPN3 cells. GAPDH was used as a loading control. MW markers are shown on the left. (**B**) RT-qPCR of the indicated EMT markers. Results are reported as relative fold change compared to HCC1569-Ctr, mean ± S.D. of four independent experiments. P value, Student's t test, two tailed. (**C**) HCC1569-Ctr and -EPN3 cells

were stimulated with 5 ng ml^{-1} of TGFβ for the indicated time points and subjected to IB analysis of the indicated EMT markers. GAPDH, loading control. MW markers are shown on the right; h.e. high exposure, l.e. low exposure. Source data are provided as a Source Data file.

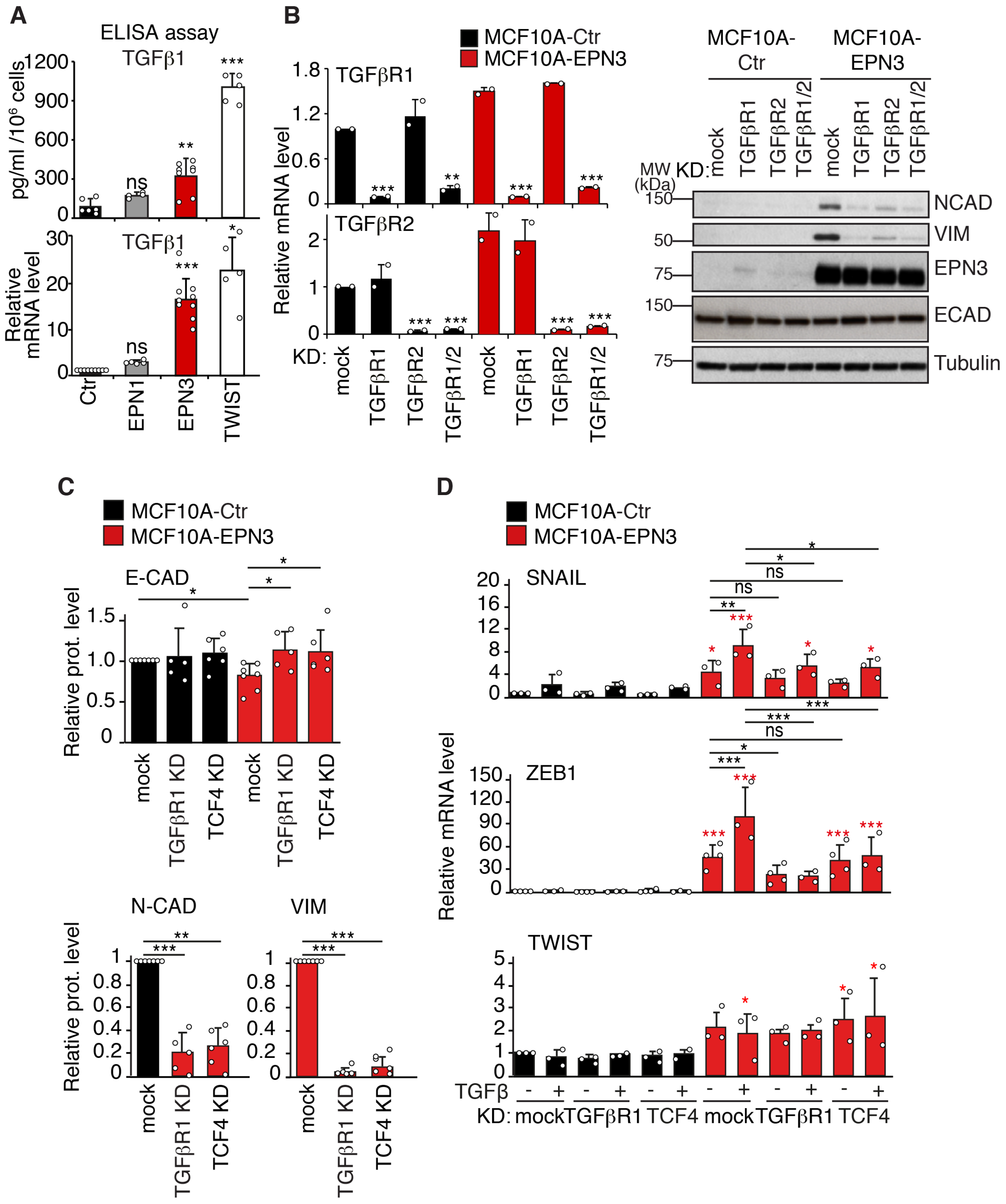

Supplementary Figure 8. Analysis of TGFβ-and TCF4-dependent phenotypes in MCF10A-EPN3 cells. (**A**) MCF10A-Ctr, -EPN1, -EPN3 and -TWIST cells were analyzed by: Top: ELISA assay of secreted TGFβ1 in conditioned media from the indicated cell cultures. Results are

reported as pg $ml^{-1}/10^6$ cells of biological replicates, mean ± S.D. (n=3 for Ctr and EPN3; n=2 for EPN1 and TWIST), each performed on the medium of at least two independent dishes, each of which was assayed in technical triplicates. P value, Student's t-test two tailed. Bottom: RT-qPCR analysis of mRNA expression of TGFβ ligand 1 (TGFβ1). Results are reported as relative mRNA expression compared to MCF10A-Ctr, mean ± S.D. of at least five independent experiments, each performed in technical triplicates. P value, Student's t-test two-tailed. In the case of MCF10A-Ctr and -EPN3 samples, the same data are also reported in Figure 6B. (**B**) MCF10A-Ctr or -EPN3 cells were transiently transfected with siRNA targeting TGFβ receptor type I (TGFβR1 KD) or type II (TGFβR2 KD) or both (TGFβR1+R2 KD), or mock transfected (see also Figure 6C-D, main text). Left, the efficiency of the silencing of TGFβRI and II was assessed by RT-qPCR analysis. Data are normalized on *GAPDH*, and are expressed as fold-change relative to the MCF10A-Ctr cells mock transfected. Results represent the mean ± S.D. of two independent experiments, each performed in technical triplicates. P values, Each Pair Student's t-test two-tailed. Right, MCF10A cells expressing the indicated constructs were analyzed by IB with the indicated Ab. Tubulin, loading control. MW markers are shown on the left. (**C**) Relative protein levels of NCAD, ECAD and VIM upon TGFβR1 and TCF4 KD shown in Figure 6C were quantified using Gel Analysis plugin on ImageJ software (v1.52, NIH), normalized on the expression of GAPDH. ECAD is reported as relative fold-change compared to Ctr mock cells; NCAD and VIM are reported as relative fold-change compared to EPN3 mock cells. Results are reported as the mean ± S.D. of independent experiments (n=5 for TGFβR1 KD samples; n=6 for TCF4 KD samples). P-values, Student's t test, two-tailed. (**D**) RT-qPCR analysis of mRNA expression of the indicated genes in MCF10A-Ctr and -EPN3 cells that were mock-treated or subjected to TGFβR1 KD or TCF4 KD, and stimulated or not with TGFβ1 (5 ng

ml^{-1} for 48 h). Results are reported as relative mRNA expression compared to the MCF10A-Ctr mock-treated sample, mean ± S.D. of at least three independent biological replicates, each performed in technical triplicates. P value, Each Pair Student's t-test two-tailed. Black asterisks indicate statistical significance between KD samples *vs.* mock, and between KD samples treated with TGFβ1 *vs.* mock treated with TGFβ1, as indicated by the bar; red asterisks indicate statistical significance between corresponding samples of MCF10A-EPN3 *vs.* Ctr cells. Source data are provided as a Source Data file.

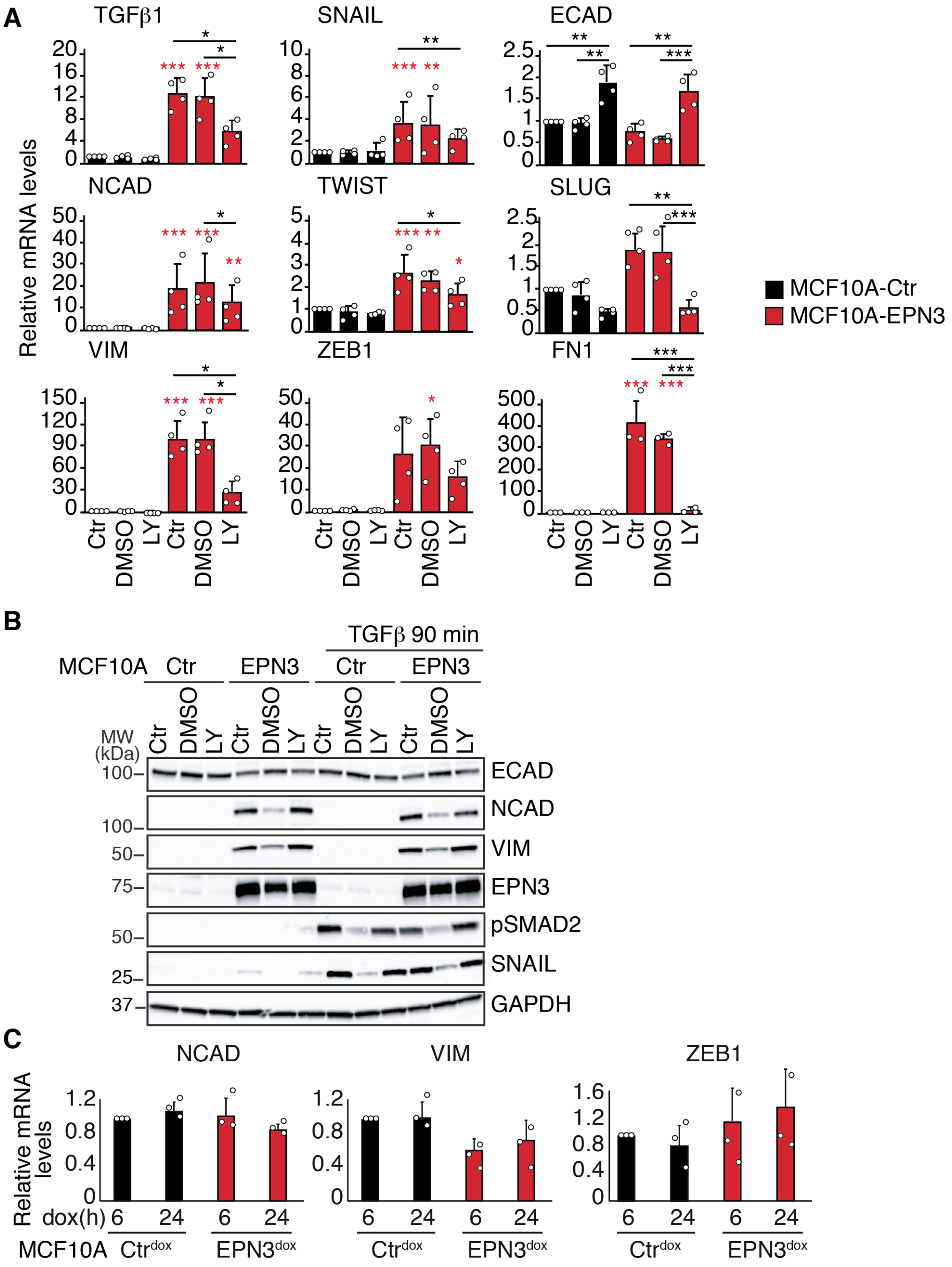

Supplementary Figure 9. Study of the relationship between EPN3- and TGFβ-dependent phenotypes. (**A**) MCF10A-Ctr or -EPN3 cells were treated with the TGFβ receptor kinase inhibitor LY2109761 (LY, 5 μM for 72 h). DMSO treatment or no treatment (Ctr) were used as negative controls. Cells were analyzed by RT-qPCR for the expression of the indicated EMT

genes. Results are reported as relative mRNA expression compared to MCF10A-Ctr (not treated), mean ± S.D. of at least three independent experiments, each performed in technical triplicates. P value, Each Pair Student's t-test two-tailed. Black asterisks indicate statistical significance between LY treated samples *vs* not treated or DMSO samples, as indicated by the bar; red asterisks indicate statistical significance between corresponding EPN3 samples *vs* Ctr. (**B**) MCF10A-Ctr or -EPN3 cells were treated as described in (A) and were stimulated for 90 min with 0.75 ng ml^{-1} TGFβ1 to check the effective inhibition of TGFβ signaling and partial reversion of the EMT phenotype by LY inhibitor by IB analysis with the indicated Ab. GAPDH, loading control. pSMAD2 and SNAIL were significantly inhibited by LY treatment. Furthermore, partial reversion of the EMT phenotype, measured as a decrease of NCAD and VIM and increase of the ECAD signal, was observed upon LY treatment in MCF10A-EPN3 cells. MW markers are shown on the left. (**C**) RT-qPCR analysis of mRNA expression of the indicated genes in MCF10A-Ctr ^{dox} and - EPN3^{dox} cells dox-induced for 6h and 24h. Results are reported as relative mRNA expression compared to MCF10A-Ctr, mean ± S.D. of three independent experiments, each performed in technical triplicates. No significant differences were scored by Each Pair, Student's t-test two-tailed. Source data are provided as a Source Data file.

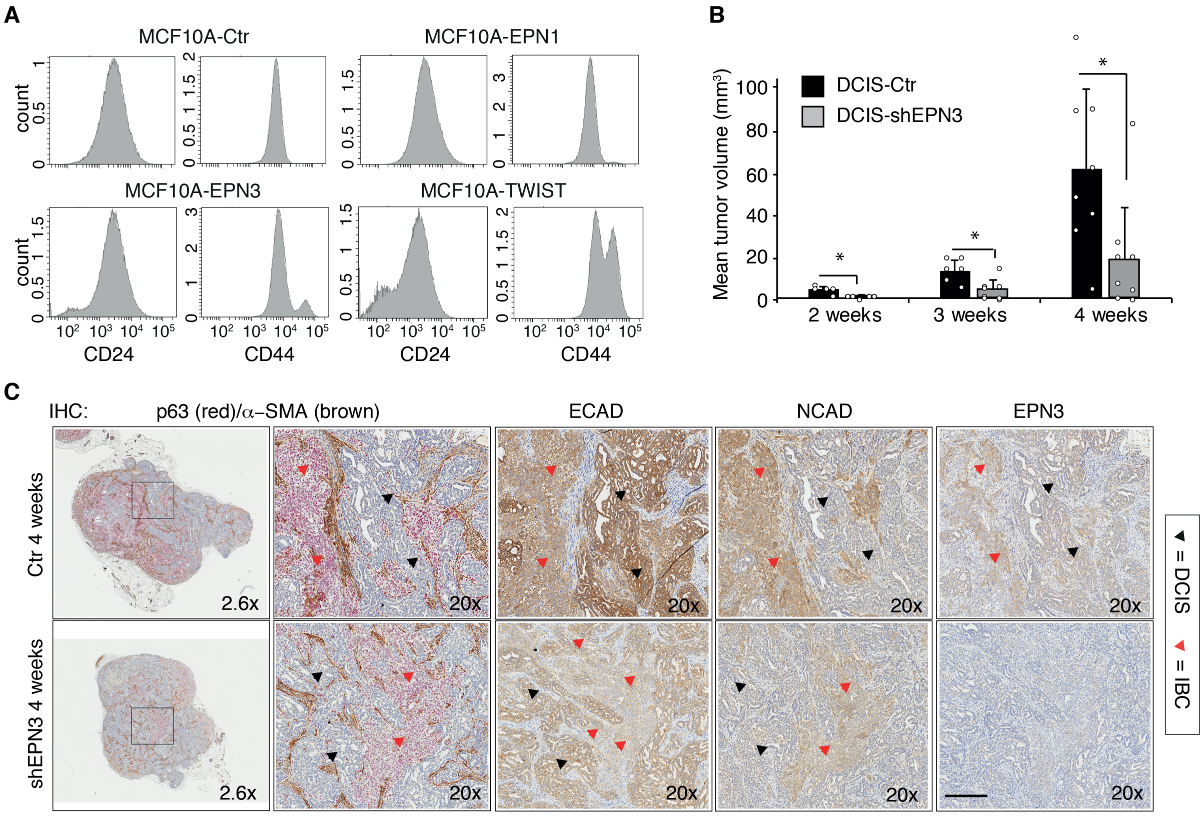

Supplementary Figure 10. Controls for bi-parametric (CD44/CD24) FACS, and for EPN3 silencing in MCF10ADCIS.com. (**A**) A representative experiment shown in Figure 8A as bi-parametric (CD44/CD24) FACS analysis is here reported as histograms of CD24 and CD44 expression, separately. Two peaks shown in EPN3 and TWIST overexpressing MCF10A cells indicate the two different populations for both CD24 and CD44 markers: a $CD24^{low}$ and $CD24^{high}$ (left); $CD44^{low}$ and $CD44^{high}$ (right). (**B**) MCF10ADCIS.com cells were infected with control vector (DCIS-Ctr) or with shEPN3 (DCIS-shEPN3) and engrafted into the mammary fat pad of female NOD/SCID IL2 gamma-chain null (NSG) mice. Tumor growth was measured as mean tumor volume at the indicated time points ± S.D. (N=19 DCIS-Ctr, N=21 DCIS-shEPN3). P value, Each Pair Student's t-test two-tailed. (**C**) Representative IHC images of *in situ* (DCIS, black arrowheads) and infiltrating (IBC, red arrowheads) areas of DCIS-Ctr and DCIS-shEPN3

tumors at 4 weeks stained with the indicated Ab on consecutive sections. One image for each tumor at 2.6X is shown on the left, while the other images are magnifications of the boxed insets, at 20X. Bar, 100 μm. Source data are provided as a Source Data file.

Supplementary Table 1. Correlation between EPN3 status determined by IHC on TMA and clinical-pathological variables in the IEO BC 97-00 cohort.

	IHC					
	LOW	HIGH	Univariate analysis		Multivariable analysis	
Variable	N (%)	N (%)	OR (95% CI)	P	OR (95% CI)	P
**All**	1133 (62.4)	684 (37.6)				
**Age at surgery**						
<50	457 (59.6)	310 (40.4)	Ref.		Ref.	
>=50	676 (64.4)	374 (35.6)	0.8 (0.9;1.0)	0.04	1.0 (0.8;1.2)	0.99
**Histology**						
Ductal	880 (58.7)	620 (41.3)	2.8 (2.1;3.7)	<.01	1.6 (1.2;2.3)	<0.01
No Ductal	253 (79.8)	64 (20.2)	Ref.		Ref.	
**pT**						
pT1a/b	123 (73.7)	44 (26.3)	0.6 (0.4;0.9)	0.01	1.5 (0.9;2.6)	0.09
pT1c	581 (60.7)	376 (39.3)	1.1 (0.9;1.3)	0.51	1.8 (1.4;2.3)	<.01
pT2	391 (62.4)	236 (37.6)	Ref.		Ref.	
pT3/pT4	38 (57.6)	28 (42.4)	1.2 (0.7;2.0)	0.45	1.3 (0.7;2.3)	0.33
**pN**						
pN0	555 (66.3)	282 (33.7)	Ref.		Ref.	
pN1-2-3	548 (58.0)	397 (42.0)	1.4 (1.2;1.7)	<.01	1.4 (1.1;1.8)	<0.01
pNX	30 (85.7)	5 (14.3)	0.3 (0.1;0.8)	0.02	0.4 (0.2;1.2)	0.10
**Grade**						
1	254 (84.1)	48 (15.9)	Ref.		Ref.	
2	543 (70.1)	232 (29.9)	2.3 (1.6;3.2)	<.01	1.5 (1.0;2.2)	0.05
3	310 (44.0)	394 (56.0)	6.7 (4.8;9.5)	<.01	3.4 (2.2;5.3)	<.01
NA	26 (72.2)	10 (27.8)	2.0 (0.9;4.5)	0.08	2.1 (0.9;4.9)	0.10
**PVI**						
Absent	782 (65.3)	415 (34.7)	Ref.		Ref.	
Present	351 (56.6)	269 (43.4)	1.4 (1.2;1.8)	<.01	1.0 (0.8;1.2)	0.82
**ER/PgR**						
Not expressed (Both 0)	116 (42.8)	155 (57.2)	2.6 (2.0;3.3)	<.01	1.3 (1.0;1.8)	0.07
Expressed (ER>0 or PgR>0)	1017 (65.8)	529 (34.2)	Ref.		Ref.	
**ERBB2 Status**						
NEG	1028 (65.6)	539 (34.4)	Ref.		Ref.	
POS	85 (38.1)	138 (61.9)	3.1 (2.3;4.1)	<.01	1.7 (1.3;2.4)	<.01
NA	20 (74.1)	7 (25.9)	0.7 (0.3;1.6)	0.36	0.5 (0.2;1.3)	0.19
**Ki-67**						
<14%	404 (83.5)	80 (16.5)	Ref.		Ref.	
>=14%	728 (54.7)	603 (45.3)	4.2 (3.2;5.4)	<.01	2.1 (1.5;2.9)	<.01
NA	1 (50.0)	1 (50.0)	5.1 (0.3;81.6)	0.25	1.7 (0.1;27.8)	0.73
**Subtype 2013**						
LUM A	258 (83.5)	51 (16.5)	Ref.		Not included	
LUM B ERBB2-NEG	677 (63.6)	388 (36.4)	2.9 (2.1;4.0)	<.01		
LUM B ERBB2-POS	52 (41.3)	74 (58.7)	7.2 (4.5;11.5)	<.01		
ERBB2-POS NO LUM	31 (34.4)	59 (65.6)	9.6 (5.7;16.3)	<.01		
TN	83 (47.2)	93 (52.8)	5.7 (3.7;8.6)	<.01		
NA	32 (62.7)	19 (37.3)	3.0 (1.6;5.7)	<.01		
**First Event**						
No event	808 (64.0)	455 (36.0)				
Loco-Regional	61 (58.1)	44 (41.9)				
Distant Metastasis	134 (50.4)	132 (49.6)				
Other	130 (71.0)	53 (29.0)				

Legend to Supplementary Table 1. IHC data of EPN3 expression were available for 1817 cases (see also Figure 10). Association between the probability of high EPN3 status and the demographic, clinical, and pathological variables was evaluated with univariate and multivariable logistic regression models and expressed as odds ratios (OR) with 95% Wald confidence intervals (CI). The number (N) of patients and percentage (%) in each group is indicated, alongside the reference (Ref.) group. Patients were assigned to EPN3 high or low group based on the following scoring systems: IHC, EPN3 low ≤1.0, EPN3 high >1.0. The event Distant Metastasis was defined as the occurrence of distant metastasis or death from BC as a first event[1]. Other events include second primary cancer or death from unknown causes or other causes. pT, primary tumor size; pN, nodal status; PVI, perivascular invasion; ER: estrogen receptor; PgR progesterone receptor; Ki-67, proliferation index. Molecular subtypes were defined according to the St. Gallen 2013 classification[2]: Luminal A, LUM A; ERBB2-negative Luminal B, LUM B ERBB2-NEG; ERBB2-positive Luminal B, LUM B ERBB2-POS; ERBB2-positive non-luminal, ERBB2-POS NO LUM; Triple-negative, TN. NA, not available. P, p-value. Source data are provided as a Source Data file.

Supplementary Table 2. Correlation between EPN3 status determined by RT-qPCR and clinical-pathological variables in the IEO BC 97-00 cohort.

	RT-qPCR					
			Univariate analysis		Multivariable analysis	
**Variable**	**LOW N (%)**	**HIGH N (%)**	**OR (95% CI)**	**P**	**OR (95% CI)**	**P**
**All**	1159 (50.0)	1160 (50.0)				
**Age at surgery**						
<50	496 (54.4)	416 (45.6)	Ref.		Ref.	
>=50	663 (47.1)	744 (52.9)	1.3 (1.1;1.6)	<.01	1.6 (1.3;1.9)	<.01
**Histology**						
Ductal	872 (46.9)	988 (53.1)	1.9 (1.5;2.3)	<.01	1.6 (1.2;2.0)	<.01
No Ductal	287 (62.5)	172 (37.5)	Ref.		Ref.	
**pT**						
pT1a/b	175 (58.7)	123 (41.3)	0.6 (0.5;0.8)	<.01	1.1 (0.8;1.5)	0.76
pT1c	595 (49.8)	600 (50.2)	0.9 (0.7;1.1)	0.20	1.1 (0.8;1.3)	0.66
pT2	350 (46.8)	398 (53.2)	Ref.		Ref.	
pT3/pT4	39 (50.0)	39 (50.0)	0.9 (0.5;1.4)	0.59	0.9 (0.5;1.4)	0.58
**pN**						
pN0	620 (55.0)	507 (45.0)	Ref.		Ref.	
pN1-2-3	511 (44.9)	626 (55.1)	1.5 (1.3;1.8)	<.01	1.2 (0.9;1.4)	0.17
pNX	28 (50.9)	27 (49.1)	1.2 (0.7;2.0)	0.55	1.3 (0.7;2.3)	0.40
**Grade**						
1	252 (58.9)	176 (41.1)	Ref.		Ref.	
2	522 (51.6)	489 (48.4)	1.3 (1.1;1.7)	0.01	1.1 (0.8;1.4)	0.60
3	354 (42.9)	472 (57.1)	1.9 (1.5;2.4)	<.01	1.7 (1.2;2.4)	<.01
NA	31 (57.4)	23 (42.6)	1.1 (0.6;1.9)	0.84	1.4 (0.8;2.6)	0.29
**PVI**						
Absent	856 (54.5)	714 (45.5)	Ref.		Ref.	
Present	303 (40.5)	446 (59.5)	1.8 (1.5;2.1)	<.01	1.4 (1.2;1.7)	<.01
**ER/PgR**						
Not expressed (Both 0)	201 (64.4)	111 (35.6)	0.5 (0.4;0.6)	<.01	0.2 (0.2;0.3)	<.01
Expressed (ER>0 or PgR>0)	958 (47.7)	1049 (52.3)	Ref.		Ref.	
**ERBB2 Status**						
NEG	927 (50.7)	902 (49.3)	Ref.		Ref.	
POS	72 (28.6)	180 (71.4)	2.6 (1.9;3.4)	<.01	3.2 (2.3;4.4)	<.01
NA	160 (67.2)	78 (32.8)	0.5 (0.4;0.7)	<.01	0.5 (0.4;0.7)	<.01
**Ki-67**						
<14%	371 (56.6)	285 (43.4)	Ref.		Ref.	
>=14%	787 (47.4)	874 (52.6)	1.5 (1.2;1.7)	<0.01	1.1 (0.8;1.3)	0.62
NA	1 (50.0)	1 (50.0)	1.3 (0.1;20.9)	0.85	1.5 (0.1;33.9)	0.78
**Subtype 2013**						
LUM A	214 (57.5)	158 (42.5)	Ref.			
LUM B ERBB2-NEG	546 (44.1)	693 (55.9)	1.7 (1.4;2.2)	<0.01		
LUM B ERBB2-POS	39 (27.1)	105 (72.9)	3.7 (2.4;5.6)	<0.01	Not included	
ERBB2-POS NO LUM	32 (32.0)	68 (68.0)	2.9 (1.8;4.6)	<0.01		
TN	157 (79.7)	40 (20.3)	0.4 (0.2;0.5)	<0.01		
NA	171 (64.0)	96 (36.0)	0.8 (0.5;1.0)	0.10		
**First Event**						
No event	866 (52.5)	782 (47.5)				
Loco-Regional	59 (45.7)	70 (54.3)				
Distant Metastasis	118 (38.1)	192 (61.9)				
Other	116 (50.0)	116 (50.0)				

Legend to Supplementary Table 2. RT-qPCR data of EPN3 expression were available for 2319 cases (see also Figure 10). Association between the probability of high EPN3 status and the demographic, clinical, and pathological variables was evaluated with univariate and multivariable logistic regression models and expressed as odds ratios (OR) with 95% Wald confidence intervals (CI). The number (N) of patients and percentage (%) in each group is indicated, alongside the reference (Ref.) group. Patients were assigned to EPN3 high or low group based on the following scoring systems: RT-qPCR (median value of Cq normalized): EPN3 low, ≥28.18, EPN3 high, <28.18 (see Methods for details). The event Distant Metastasis was defined as the occurrence of distant metastasis or death from BC as a first event[1]. Other events include second primary cancer or death from unknown causes or other causes. pT, primary tumor size; pN, nodal status; PVI, perivascular invasion; ER: estrogen receptor; PgR progesterone receptor; Ki-67, proliferation index. Molecular subtypes were defined according to the St. Gallen 2013 classification[2]: Luminal A, LUM A; ERBB2-negative Luminal B, LUM B ERBB2-NEG; ERBB2-positive Luminal B, LUM B ERBB2-POS; ERBB2-positive non-luminal, ERBB2-POS NO LUM; Triple-negative, TN. NA, not available. P, p-value. Source data are provided as a Source Data file.

Supplementary Table 3. Prognostic value of EPN3 status, determined by IHC and RT-qPCR analysis, in different subgroups of the IEO BC 97-00 cohort.

		Distant Metastasis events				Loco-Regional events			
		Univariate analysis		Multivariable analysis		Univariate analysis		Multivariable analysis	
	Subgroups	HR (95% CI) HIGH vs LOW	P	HR (95% CI) HIGH vs LOW	P	HR (95% CI) HIGH vs LOW	P	HR (95% CI) HIGH vs LOW	P
IHC	All patients	1.7 (1.4;2.2)	<.01	1.3 (1.0;1.7)	0.03	1.3 (0.9;1.9)	0.23	1.0 (0.7;1.5)	0.98
	ERBB2-NEG	1.8 (1.4;2.4)	<.01	1.4 (1.0;1.8)	0.02	1.3 (0.8;2.0)	0.23	1.1 (0.7;1.8)	0.60
	ERBB2-POS	0.9 (0.5;1.6)	0.71	0.9 (0.4;1.6)	0.63	0.7 (0.3;1.6)	0.35	0.6 (0.2;1.4)	0.19
	pN0	2.4 (1.5;3.8)	<.01	1.6 (1.0;2.6)	0.05	1.0 (0.5;1.9)	0.90	0.8 (0.4;1.6)	0.47
	pN1-2-3	1.4 (1.0;1.8)	0.03	1.2 (0.9;1.6)	0.24	1.3 (0.8;2.1)	0.31	1.1 (0.6;1.8)	0.83
	LUM A	1.2 (0.3;4.0)	0.83	1.0 (0.3;3.7)	0.99	0.6 (0.1;4.9)	0.64	0.6 (0.1;5.2)	0.65
	LUM B ERBB2-NEG	1.7 (1.3;2.3)	<.01	1.4 (1.0;1.9)	0.03	1.2 (0.7;2.0)	0.44	1.1 (0.7;1.9)	0.65
	LUM B ERBB2-POS	1.0 (0.5;2.1)	0.97	0.9 (0.4;2.1)	0.88	0.5 (0.1;2.0)	0.35	0.5 (0.1;1.9)	0.30
	ERBB2-POS NO LUM	0.7 (0.2;2.1)	0.53	0.6 (0.2;1.8)	0.36	0.7 (0.2;2.3)	0.58	0.6 (0.2;2.2)	0.48
	TN	1.3 (0.6;2.7)	0.46	1.4 (0.6;3.3)	0.38	1.2 (0.3;4.4)	0.81	1.4 (0.3;6.8)	0.69
RT-qPCR	All patients	1.7 (1.4;2.2)	<.01	1.4 (1.1;1.7)	0.01	1.3 (0.9;1.8)	0.19	1.1 (0.8;1.6)	0.64
	ERBB2-NEG	1.7 (1.3;2.2)	<.01	1.5 (1.2;2.0)	<.01	1.3 (0.8;1.9)	0.25	1.2 (0.8;1.8)	0.38
	ERBB2-POS	0.8 (0.4;1.5)	0.53	0.8 (0.4;1.5)	0.54	0.7 (0.3;1.7)	0.42	0.6 (0.3;1.5)	0.31
	pN0	2.4 (1.5;3.9)	<.01	2.3 (1.4;3.9)	<.01	0.8 (0.5;1.5)	0.58	0.8 (0.4;1.4)	0.39
	pN1-2-3	1.3 (1.0;1.7)	0.05	1.2 (0.9;1.5)	0.31	1.4 (0.9;2.2)	0.18	1.3 (0.8;2.1)	0.28
	LUM A	1.4 (0.6;3.4)	0.45	1.4 (0.5;3.4)	0.53	0.6 (0.2;2.3)	0.47	0.6 (0.2;2.5)	0.52
	LUM B ERBB2-NEG	1.8 (1.3;2.5)	<.01	1.6 (1.1;2.1)	<.01	1.3 (0.8;2.1)	0.21	1.3 (0.8;2.1)	0.23
	LUM B ERBB2-POS	1.0 (0.4;2.1)	0.91	1.0 (0.5;2.2)	0.96	0.5 (0.1;1.5)	0.19	0.4 (0.1;1.4)	0.15
	ERBB2-POS NO LUM	0.7 (0.3;1.8)	0.43	0.7 (0.2;1.9)	0.46	1.3 (0.3;4.7)	0.73	1.1 (0.3;4.5)	0.89
	TN	1.3 (0.5;2.9)	0.59	1.5 (0.6;3.6)	0.34	2.0 (0.6;6.5)	0.25	1.8 (0.5;6.2)	0.38

Legend to Supplementary Table 3. The hazard ratios (HR) of Distant Metastasis or Loco-Regional events in the comparison of EPN3 high *vs*. low tumors were estimated with both univariate and multivariable Cox proportional hazard models (see also Figure 10). Multivariable

models were adjusted for Grade, Ki-67, ERBB2 status, estrogen/progesterone status, tumor size, number of positive lymph nodes and age at surgery (as appropriate in each subgroup analysis). Patients were assigned to EPN3 high or low groups based on the following scoring systems: IHC, EPN3 low ≤1.0, EPN3 high >1.0; RT-qPCR (median value of Cq normalized): EPN3 low ≥28.18, EPN3 high <28.18 (see Materials and Methods for details). The number of patients in each subgroup analysis corresponds to the ones reported in Tables S1 and S2 for IHC and RT-qPCR, respectively. pN, nodal status. Molecular subtypes were defined according to the St. Gallen 2013 classification[2]: Luminal A, LUM A; ERBB2-negative Luminal B, LUM B ERBB2-NEG; ERBB2-positive Luminal B, LUM B ERBB2-POS; ERBB2-positive non-luminal, ERBB2-POS NO LUM; Triple-negative, TN. CI, 95% Wald confidence intervals; P, p-value. Source data are provided as a Source Data file.

Supplementary Table 4. Primary antibodies used in the study.

Primary Antibody	Source	Dilution
anti-EPN3	Mouse monoclonal, homemade (VI31, epitope: aa residues 464-483, Homo sapiens)[3]	1:1000 for IB<br>1:5000 for IF<br>1:30000 for IHC
anti-EPN3	Rabbit polyclonal, homemade (VI31, epitope: aa residues 464-483, Homo sapiens)[3]	1:1000 for IB<br>1:5000 for IF and PLA<br>1:30000 for IHC<br>5 ng/mg lysate for CoIP
anti-EPN1/2	Mouse monoclonal, homemade (ZZ3, epitope: aa residues 249-401)	1:500 for IB
anti-vinculin	Sigma, clone hVIN-1, V9131	1:5000 for IB
anti-tubulin	Sigma, clone DM1A, T9026	1:5000 for IB
anti-Flag	Sigma, clone M2, F3165	1:1000 for IB<br>1:5000 for IF
anti-clathrin heavy chain	BD Bioscience, clone 23, 610499	1:1000 for IB
anti-ECAD (intracellular domain)	BD Bioscience, clone 36, 610181	1:1000 for IB<br>1:200 for IF<br>1:200 for PLA<br>1:1500 for IHC
anti-ECAD (extracellular domain)	Abcam, HECD-1, ab1416	1:25 for internalization assay<br>for FACS and IF
anti-NCAD	BD Bioscience, clone 32, 310920	1:1000 for IB
anti-NCAD	Dako, clone 6G11, M3613	1:50 for IHC
anti-VIM	BD Bioscience, clone RV202, 550513	1:1000 for IB
anti-SNAIL	Cell Signaling, L70G2, 3895	1:1000 for IB
anti-pSMAD2	Cell Signaling, 138D4, 3108	1:1000 for IB
anti-pSMAD3	Cell Signaling, C25A9, 9520	1:1000 for IB
anti-TWIST	Santa Cruz, 2C1a, sc-81417	1:500 for IB
Anti-c-ErbB2	Dako, A0485	1:1000 for IHC
anti-active-beta-catenin	Millipore, 8E7, 05-665	1:200 for IF
anti-epiligrin (Laminin 5)	Millipore, P3H9-2, MAB1947	1:200 for IF
anti-giantin	Biolegend, Poly19243, 924301	1:200 for IF
anti-keratin-5	Abcam, ab53121	1:100 for IF
anti-keratin-8	Abcam, ab53280	1:100 for IF
anti-keratin-14	Abcam, ab7800	1:100 for IF
anti-CD44	BD Bioscience, clone G44-26, APC-conjugated	1:5 for FACS
anti-CD24	BD Bioscience, clone ML5, PE-conjugated	1:5 for FACS
anti-p63	Abcam, EPR5701, ab124762	1:8000 for IHC
anti-alpha-SMA	Dako, 1A4, M0851	1:200 for IHC

Legend to Supplementary Table 4. The table describes the primary antibodies used in the study, with name, source, clone and catalog number and dilution used in the different assays (IB, immunoblot; IF, immunofluorescence; PLA, proximity ligation assay; IHC, immunohistochemistry; CoIP, co-immunoprecipitation; FACS, fluorescence-activated cell sorting).

Supplementary Table 5. Secondary antibodies used in the study.

**Secondary Antibody**	**Source**	**Dilution**
anti-rabbit IgG HRP-linked	Cell Signaling, 7074	1:2000 for IB
anti-mouse IgG HRP-linked	Cell Signaling, 7076	1:2000 for IB
Alexa Fluor 488 donkey Anti-rabbit IgG	Thermo Fisher, A-21206	1:200 for Matrigel IF<br>1:400 for all other IF
Alexa Fluor 488 donkey Anti-mouse IgG	Thermo Fisher, A-21202	1:200 for Matrigel IF<br>1:400 for all other IF
Alexa Fluor 647 donkey Anti-rabbit IgG	Thermo Fisher, A-31573	1:200 for Matrigel IF<br>1:400 for all other IF
Alexa Fluor 488 donkey Anti-mouse IgG	Thermo Fisher, A-31571	1:200 for Matrigel IF<br>1:400 for all other IF
Cy3 donkey Anti-rabbit IgG	Jackson ImmunoResearch, 715-165-152	1:200 for Matrigel IF<br>1:400 for all other IF
Cy3 donkey Anti-mouse IgG	Jackson ImmunoResearch, 715-165-150	1:200 for Matrigel IF<br>1:400 for all other IF

Legend to Supplementary Table 5. The table describes the secondary antibodies used in the study, with name, source, catalog number and dilution used in the different assays (IB, immunoblot; IF, immunofluorescence; Matrigel IF, immunofluorescence on matrigel-grown organoids).

Supplementary Table 6. List of primers used in the study.

Gene	TaqMan Ref or Sequence	Aim / Purpose
EPN3	For: GGAATTCTCAGAGGAAGGGGTTGGTGC<br>Rev: GAAGATCTACGACCTCCGCACTCCGG	Constructs and plasmids
EPN3	Hs00978957_m1	Quantitative real-time PCR analysis of human BCs
HPRT1	Hs02800695_m1	Quantitative real-time PCR analysis of human BCs
GAPDH	Hs03929097_g1	Quantitative real-time PCR analysis of human BCs
GUSB	Hs99999908_m1	Quantitative real-time PCR analysis of human BCs
TBP	Hs00427621_m1	Quantitative real-time PCR analysis of human BCs
CDH1	Hs00170423_m1	Quantitative real-time PCR analysis
CDH2	Hs00169953_m1	Quantitative real-time PCR analysis
VIM	Hs00185584_m1	Quantitative real-time PCR analysis
TGFB1	Hs00998133_m1	Quantitative real-time PCR analysis
TGFB2	Hs00234244_m1	Quantitative real-time PCR analysis
TGFBR1	Hs00610320_m1	Quantitative real-time PCR analysis
TGFBR2	Hs00234253_m1	Quantitative real-time PCR analysis
ZEB1	Hs00232783_m1	Quantitative real-time PCR analysis
ACTB	Hs99999903_m1	Quantitative real-time PCR analysis
18S	Hs99999901_s1	Quantitative real-time PCR analysis
GAPDH	Hs99999905_m1	Quantitative real-time PCR analysis
EPN3	Hs00978957_m1	EPN3 transcripts and copy number analysis in breast cell lines
GAPDH	Hs03929097_g1	EPN3 transcripts and copy number analysis in breast cell lines

ERBB2	For: GGATGTGCGGCTCGTACAC<br>Rev: TAATTTTGACATGGTTGGGACTCTT<br>Probe-FAM: ACTTGGCCGCTCGG	EPN3 transcripts and copy number analysis in breast cell lines
CEP17	For: TTGCAGCACGTGGCACAT<br>Rev: ACGGCAGCAAGAGAGGAAAG<br>Probe-FAM: CACTGCCTGAGCACC	EPN3 transcripts and copy number analysis in breast cell lines

Legend to Supplementary Table 6. The table indicates the primers used in the study, with name, TaqMan reference number or sequence (for custom-designed primers), aim and purpose of their use.

REFERENCES TO SUPPLEMENTARY INFORMATION

1. Hudis, C.A. *et al.* Proposal for standardized definitions for efficacy end points in adjuvant breast cancer trials: the STEEP system. *Journal of clinical oncology : official journal of the American Society of Clinical Oncology* **25**, 2127-2132 (2007).

2. Goldhirsch, A. *et al.* Personalizing the treatment of women with early breast cancer: highlights of the St Gallen International Expert Consensus on the Primary Therapy of Early Breast Cancer 2013. *Ann Oncol* **24**, 2206-2223 (2013).

3. Ko G*, et al.* Selective high-level expression of epsin 3 in gastric parietal cells, where it is localized at endocytic sites of apical canaliculi. *Proceedings of the National Academy of Sciences of the United States of America* **107**, 21511-21516 (2010).
