# Supplementary material for: A self-sustaining endocytic-based loop promotes breast cancer plasticity leading to aggressiveness and pro-metastatic behavior
Source: Nat Commun. 2020 Jun 15;11:3020. doi: 10.1038/s41467-020-16836-y (PMC7296024; doi:10.1038/s41467-020-16836-y)
Supplement: Supplementary file 3 — Reporting Summary [file 41467_2020_16836_MOESM3_ESM.pdf]

nature research

Corresponding author(s): Pier Paolo Di Fiore, Sara Sigismund, Manuela Vecchi

Last updated by author(s): Feb 4, 2020

Reporting Summary

Nature Research wishes to improve the reproducibility of the work that we publish. This form provides structure for consistency and transparency in reporting. For further information on Nature Research policies, see Authors & Referees and the Editorial Policy Checklist.

Statistics

For all statistical analyses, confirm that the following items are present in the figure legend, table legend, main text, or Methods section.

n/a	Confirmed	
☐	☒	The exact sample size (*n*) for each experimental group/condition, given as a discrete number and unit of measurement
☐	☒	A statement on whether measurements were taken from distinct samples or whether the same sample was measured repeatedly
☐	☒	The statistical test(s) used AND whether they are one- or two-sided<br>*Only common tests should be described solely by name; describe more complex techniques in the Methods section.*
☐	☒	A description of all covariates tested
☐	☒	A description of any assumptions or corrections, such as tests of normality and adjustment for multiple comparisons
☐	☒	A full description of the statistical parameters including central tendency (e.g. means) or other basic estimates (e.g. regression coefficient) AND variation (e.g. standard deviation) or associated estimates of uncertainty (e.g. confidence intervals)
☐	☒	For null hypothesis testing, the test statistic (e.g. *F*, *t*, *r*) with confidence intervals, effect sizes, degrees of freedom and *P* value noted<br>*Give P values as exact values whenever suitable.*
☒	☐	For Bayesian analysis, information on the choice of priors and Markov chain Monte Carlo settings
☒	☐	For hierarchical and complex designs, identification of the appropriate level for tests and full reporting of outcomes
☐	☒	Estimates of effect sizes (e.g. Cohen's *d*, Pearson's *r*), indicating how they were calculated

Our web collection on statistics for biologists contains articles on many of the points above.

Software and code

Policy information about availability of computer code

Data collection	-IHC images were collected with Aperio ScanScope (Leica).<br>-Immuno Blotting acquisitions were performed with Image Lab software (Bio-Rad Laboratories).<br>-Immunofluorence data was collected with Las X (Leica).<br>-FACS data were collected with FACSDiva Software (BD).<br>- RT-qPCR data were collected with 7500 Software version 2.0.6 (Applied Byosistems) or with LitghtCylcer480 (Roche).<br>-Gene amplification data were collected on TCGA website and on METABRIC website as indicated in figure legend.
Data analysis	-IHC images were analysed with Aperio ScanScope (Leica) and/or ImageJ.<br>-Immuno Blotting analysis was performed with Image Lab software (Bio-Rad Laboratories) and/or Image J.<br>-Immunofluorence analysis was performed with Image J.<br>-FACS data was analysed with BD FACSDiva Software and Flow Jo (BD).<br>-Statistical analysis of RT-qPCR experiments, IB and IF quantifications, FACS analysis, ELISA assay, Mammosphere assay, radioactive internalization experiments, Matrigel morphogenetic assay, soft agar growth and invasion assays were performed with Excel software or with JMP software (SAS Institute).<br>-Clinical data were analysed with SAS software (SAS Institute) or JMP software (SAS Institute).<br>See Methods for details.

For manuscripts utilizing custom algorithms or software that are central to the research but not yet described in published literature, software must be made available to editors/reviewers. We strongly encourage code deposition in a community repository (e.g. GitHub). See the Nature Research guidelines for submitting code & software for further information.

Data

Policy information about availability of data

All manuscripts must include a data availability statement. This statement should provide the following information, where applicable:

- Accession codes, unique identifiers, or web links for publicly available datasets
- A list of figures that have associated raw data
- A description of any restrictions on data availability

All data are available in the manuscript, in the Supplementary Materials or in Source data files.
Figures with associated raw data: Fig. 1-9 and Fig. S1-S9 and Table S1-S3

Field-specific reporting

Please select the one below that is the best fit for your research. If you are not sure, read the appropriate sections before making your selection.

☒ Life sciences ☐ Behavioural & social sciences ☐ Ecological, evolutionary & environmental sciences

For a reference copy of the document with all sections, see nature.com/documents/nr-reporting-summary-flat.pdf

Life sciences study design

All studies must disclose on these points even when the disclosure is negative.

ample size	No sample size calculations were performed; the number of experiments (n) is indicated in each figure legend.
Data exclusions	From the IEO BC 97-00 cohort of 2,453 samples, 2,275 were appropriate for the construction of ad hoc tissue microarrays (TMAs): samples with massive inflammatory infiltration, massive necrosis, minimal areas of infiltrating carcinoma were discarded. For the RT-qPCR analysis, 2,335 tissue blocks were appropriate for RNA extraction; 16 samples (0.7%) were excluded from the analysis shown in Table S2 and in Fig. 9C,D, either for lack of sufficient RNA or because of spurious RT-qPCR results (due to poor quality mRNA). Therefore, 2319 patients (99.3%) were analyzed by RT-qPCR.<br>From the IEO BC 97-00 cohort of 2,453 samples, 1817 samples were appropriate for IHC data shown in Table S1 and in Fig. 9C,D.(79.9%), mainly due to loss of tissue cores during staining and/or lack of neoplastic tissue in the tissue core.
Replication	The number of experiments replicated (n) is indicated in each figure legend.
Randomization	Samples were randomly assigned.
linding	N/A

Reporting for specific materials, systems and methods

We require information from authors about some types of materials, experimental systems and methods used in many studies. Here, indicate whether each material, system or method listed is relevant to your study. If you are not sure if a list item applies to your research, read the appropriate section before selecting a response.

Materials & experimental systems

n/a	Involved in the study	
☐	☒	Antibodies
☐	☒	Eukaryotic cell lines
☒	☐	Palaeontology
☐	☒	Animals and other organisms
☒	☐	Human research participants
☐	☒	Clinical data

Methods

n/a	Involved in the study	
☒	☐	ChIP-seq
☐	☒	Flow cytometry
☒	☐	MRI-based neuroimaging

Antibodies

ntibodies used	Primary Ab:<br>-homemade mouse monoclonal and rabbit polyclonal antibodies anti-EPN3 (VI31, epitope: aa residues 464-483, Homo sapiens). Dilutions used were 1:1000 for IB, 1:5000 for IF and PLA assay and 1:30000 IHC. 5ng of anti-rabbit EPN3 antibody/mg of lysate was used in CoIP experiments.<br>- homemade anti- mouse monoclonal EPN1/2 (ZZ3, epitope: aa 249-401, Homo sapiens. 1:500 for IB.<br>-anti-vinculin, Sigma, clone hVIN-1, V9131. 1:5000 for IB.

-anti-tubulin, Sigma, clone DM1A, T9026. 1:5000 for IB.
-anti-Flag, Sigma, clone M2, F3165. 1:1000 for IB, 1:5000 for IF.
-anti-clathrin heavy chain, BD Bioscience, clone 23, 610499. 1:1000 for IB
-anti-ECAD, BD Bioscience, clone 36, 610181. Dilutions used were 1:1000 for IB, 1:200 for IF, 1:200 for PLA assay, 1:1500 for IHC.
-anti-ECAD mouse monoclonal, Abcam, HECD-1, ab1416. 1:25 for internalization assays both for FACS and IF analysis.
-anti-NCAD, BD Bioscience, clone 32, 610920. 1:1000 for IB.
-anti-NCAD, Dako, clone 6G11, M3613, 1:50 for IHC.
-anti-VIM, BD Bioscience, clone RV202, 550513. 1:1000 for IB.
-anti-SNAIL, Cell Signaling, L70G2, 3895. 1:1000 for IB.
-anti-pSMAD2, Cell Signaling, 138D4, 3108. 1:1000 for IB.
-anti-TWIST, Santa Cruz, 2C1a, sc-81417. 1:500 for IB.
-anti-active beta-catenin, Millipore, 8E7, 05-665. 1:200 for IF.
-anti epiligrin (Laminin 5), Millipore, P3H9-2, MAB1947. 1:200 for IF.
-anti-giantin, Biolegend, Poly19243, 924301. 1:200 for IF.
-anti-keratin-5, Abcam, ab53121. 1:100 for IF.
-anti-keratin-8, Abcam, ab53280. 1:100 for IF.
-anti-keratin-14, Abcam, ab7800. 1:100 for IF.
-anti-CD44, BD Bioscience, clone G44-26, APC-conjugated. 1:5 for FACS.
-anti-CD24, BD Bioscience, clone ML5, PE-conjugated. 1:5 for FACS.
-anti-p63, Abcam, EPR5701, ab124762, 1:8000 for IHC.
-anti-alpha-SMA Dako, 1A4, M0851, 1:200 for IHC.

Secondary Ab:
-anti-rabbit IgG HRP-linked, Cell Signaling, 7074. 1:2000 for IB.
-anti-mouse IgG HRP-linked, Cell Signaling, 7076. 1:2000 for IB.

-Alexa Fluor 488 donkey anti rabbit IgG, Thermo Fisher, A-21206. 1:400 for IF.
-Alexa Fluor 488 donkey anti mouse IgG, Thermo Fisher, A-21202. 1:400 for IF.
-Alexa Fluor 647 donkey anti rabbit IgG, Thermo Fisher, A-31573. 1:400 for IF.
-Alexa Fluor 647 donkey anti mouse IgG, Thermo Fisher, A-31571.1:400 for IF.
-Cy3 donkey anti rabbit IgG, Jackson ImmunoResearch,715-165-152.1:400 for IF.
-Cy3 donkey anti mouse IgG, Jackson ImmunoResearch, 715-165-150.1:400 for IF.

Validation

Primary Ab:
Homemade antibodies were validated in house through RNA interference and IB, IF and/or IHC experiments. Anti-EPN3 antibodies were already described (Spradling KD et al., 2001; Ko G. et al., PNAS 2010).
Validation statements available from manufacturers:
anti-vinculin: https://www.sigmaaldrich.com/catalog/product/SIGMA/V9131?lang=it&region=IT
anti-tubulin: https://www.sigmaaldrich.com/catalog/product/sigma/t9026?lang=it&region=IT
Anti-FLAG: https://www.sigmaaldrich.com/catalog/product/sigma/f3165?lang=it&region=IT
Anti- clathrin heavy chain: https://www.bdbiosciences.com/eu/reagents/research/antibodies-buffers/cell-biology-reagents/cell-biology-antibodies/purified-mouse-anti-clathrin-heavy-chain-23clathrin-heavy-chain/p/610499
Anti-ECAD BD: https://www.bdbiosciences.com/eu/applications/research/stem-cell-research/cancer-research/human/purified-mouse-anti-e-cadherin-36e-cadherin/p/610181
Anti-ECAD Abcam: https://www.abcam.com/e-cadherin-antibody-hecd-1-intercellular-junction-marker-ab1416.html
Anti-NCAD BD: https://www.bdbiosciences.com/us/applications/research/stem-cell-research/cancer-research/human/purified-mouse-anti-n-cadherin-32n-cadherin/p/610920
Anti-NCAD DAKO: https://www.citeab.com/antibodies/2414755-m3613-n-cadherin
Anti-VIM BD: https://www.bdbiosciences.com/eu/applications/research/stem-cell-research/cancer-research/human/purified-mouse-anti-vimentin-rv202/p/550513
Anti-SNAIL: https://www.cellsignal.com/products/primary-antibodies/snail-l70g2-mouse-mab/3895
Anti-pSMAD2: https://www.cellsignal.com/products/primary-antibodies/phospho-smad2-ser465-467-138d4-rabbit-mab/3108
Anti-TWIST: https://www.scbt.com/p/twist-antibody-twist2c1a
Anti-active-beta-catenin: https://www.merckmillipore.com/IT/it/product/Anti-Active-Catenin-Anti-ABC-Antibody-clone-8E7,MM_NF-05-665
Anti-epiligrin, Laminin 5: https://www.merckmillipore.com/IT/it/product/Anti-Laminin-5-Antibody-clone-P3H9-2,MM_NF-MAB1947?ReferrerURL=https%3A%2F%2Fwww.google.com%2F&bd=1
Anti-giantin: https://www.biolegend.com/en-us/products/anti-giantin-antibody-11064
Anti-keratin-5: https://www.abcam.com/cytokeratin-5-antibody-cytoskeleton-marker-ab53121.html
Anti-keratin-8: https://www.abcam.com/cytokeratin-8-antibody-ep1628y-cytoskeleton-marker-ab53280.html
Anti-keratin-14: https://www.abcam.com/cytokeratin-14-antibody-ll002-ab7800.html
Anti-CD44: https://www.bdbiosciences.com/us/applications/research/t-cell-immunology/t-follicular-helper-tfh-cells/surface-markers/human/apc-mouse-anti-human-cd44-g44-26-also-known-as-c26/p/559942
Anti-CD24: https://www.bdbiosciences.com/us/applications/research/stem-cell-research/pluripotent-stem-cell-markers-esc-and-ipsc/human/pe-mouse-anti-human-cd24-ml5/p/560991
Anti-p63: https://www.abcam.com/p63-antibody-epr5701-ab124762.html
Anti-alpha-SMA: https://www.labome.com/product/Dako/M0851.html

Secondary Ab:
Anti-IgG RABBIT HRP: https://www.cellsignal.com/products/secondary-antibodies/anti-rabbit-igg-hrp-linked-antibody/7074
Anti-IgG MOUSE HRP: https://www.cellsignal.com/products/secondary-antibodies/anti-mouse-igg-hrp-linked-antibody/7076
Anti-Alexa Fluor 488 DONKEY ANTI MOUSE:https://www.thermofisher.com/antibody/product/Donkey-anti-Mouse-IgG-H-L-Highly-Cross-Adsorbed-Secondary-Antibody-Polyclonal/A-21202
Anti-Alexa Fluor 488 DONKEY ANTI RABBIT: https://www.thermofisher.com/antibody/product/Donkey-anti-Rabbit-IgG-H-L-

Highly-Cross-Adsorbed-Secondary-Antibody-Polyclonal/A-21206
Anti-Alexa Fluor 647 DONKEY ANTI MOUSE: https://www.thermofisher.com/antibody/product/Donkey-anti-Mouse-IgG-H-L-Highly-Cross-Adsorbed-Secondary-Antibody-Polyclonal/A-31571
Anti-Alexa Fluor 647 DONKEY ANTI RABBIT: https://www.thermofisher.com/antibody/product/Donkey-anti-Rabbit-IgG-H-L-Highly-Cross-Adsorbed-Secondary-Antibody-Polyclonal/A-31573
-Cy3 donkey anti rabbit IgG, https://www.jacksonimmuno.com/catalog/products/711-165-152
-Cy3 donkey anti mouse IgG, https://www.jacksonimmuno.com/catalog/products/715-165-150

Eukaryotic cell lines

Policy information about cell lines

ell line source(s)
All human breast cell lines were from the American Type Culture Collection (ATCC), with the exception of MCF10ADCIS.com, which were kindly provided by Dr. John F Marshall (Barts Cancer Institute, London, UK).

Authentication
All human cell lines were authenticated at each batch freezing by STR profiling (StemElite ID System, Promega).

ycoplasma contamination
All human cell lines were tested for mycoplasma by PCR (Uphoff and Drexler, 2002) and biochemical assay (MycoAlert, Lonza).

Commonly misidentified lines (See ICLAC register)
NO

Animals and other organisms

Policy information about studies involving animals; ARRIVE guidelines recommended for reporting animal research

aboratory animals
Female mice between 6 and 12 weeks old were used for all experiments. Mouse lines used: EPN3-KI in FVB background, NOD/SCID IL2R gamma-chain null (NSG).

Wild animals
This study did not involve wild animals.

Field-collected samples
This study did not involve animals collected from the field.

Ethics oversight
All mouse experiments were conducted in a certified animal facility under the control of the institutional organism for animal welfare and ethical approach to animals in experimental procedures (Cogentech OPBA). All animal studies were conducted with the approval of Italian Minister of Health (27/2015-PR) and were performed in accordance with the Italian law (D.lgs. 26/2014), which enforces Dir. 2010/63/EU (Directive 2010/63/EU of the European Parliament and of the Council of 22 September 2010 on the protection of animals used for scientific purposes).

Note that full information on the approval of the study protocol must also be provided in the manuscript.

Clinical data

Policy information about clinical studies
All manuscripts should comply with the ICMJE guidelines for publication of clinical research and a completed CONSORT checklist must be included with all submissions.

Clinical trial registration
A cohort of BC patients derived from a clinical study (Trial registration ID: NCT00970983, Veronesi, U. et al. N Engl J Med 349, 546-553, 2003) was employed for the FISH analysis (Figure 1B).

tudy protocol
The cohort used for IHC and RT-qPCR analyses shown in Figures 9C,D and Tables S1-S3 was a retrospective consecutive cohort of patients enrolled at IEO between years 1997-2000. Detailed description of the selection criteria and the clinico-pathological characteristics of this cohort have been reported in (Pece, S. et al., EBioMedicine 2019).

Data collection
- The FISH analysis (Figure 1B) was performed on a cohort of BC patients derived from a clinical study (Trial registration ID: NCT00970983, Veronesi, U. et al. N Engl J Med 349, 546-553, 2003), for which TMAs were already available in the lab and previously described (Colaluca, I.N. et al. Nature 451, 76-80, 2008; Confalonieri, S. et al. Oncogene 28, 2959-2968, 2009).
- For the cohort used for IHC and RT-qPCR, available clinical and pathological information included age, date at surgery, tumor characteristics (histological type, tumor size, nodal involvement, grade, perivascular infiltration, Ki-67 and ER/PgR expression) and treatment modality (type of surgery, adjuvant radiotherapy, endocrine therapy, chemotherapy). Patients of this cohort were followed up with physical examination every 6 months, annual mammography and breast ultrasound, blood tests every 6-12 months and further evaluations only in case of symptoms. When possible, the status of women not presenting at the institute for scheduled follow-up visits for more than one year was obtained by telephone contact.

Outcomes
Cumulative incidence of Loco-regional (LR) and Distant Metastasis (CI-DM) were defined as the time from surgery to the appearance of a local or regional recurrence and distant metastases or death from BC as first event, respectively. Second primary cancer or death from unknown causes or other causes were considered competing events. Considering first events,

median follow-up for censored patients was 14.1 years (interquartile range [IQR] 12.1-15.7). One hundred and eighty-five (7.5%) patients were lost at 10 years of follow-up.

Flow Cytometry

Plots

Confirm that:

☒ The axis labels state the marker and fluorochrome used (e.g. CD4-FITC).

☒ The axis scales are clearly visible. Include numbers along axes only for bottom left plot of group (a 'group' is an analysis of identical markers).

☒ All plots are contour plots with outliers or pseudocolor plots.

☒ A numerical value for number of cells or percentage (with statistics) is provided.

Methodology

Sample preparation	Live cells were incubated with ECAD, Abcam, antibody for 1h at 4C and then incubated with Alexa Fluor 488 donkey anti-mouse for 30 min at 4C. Then cells were treated or not and then trypsinized and fixed. See Methods for details.<br>For CD24 and CD44 double staining cells were washed with PBS, trypsinized, stained and then fixed. See Methods for details.
Instrument	BD FACS Canto II or BD FACSAria or BD FACS Celesta.
Software	FACS data were collected with FACSDiva Software (BD) and analyzed with FACSDiva Software (BD) or Flow Jo (BD).
Cell population abundance	ECAD plasma membrane positive and negative populations are shown in Fig 3B and S3B with relative abundance. ECAD internalized population is shown Fig 3C, 4B, 4F, 6E. Coherent data have been obtained by IF.<br>CD44/CD24 populations are shown in Fig 7A, S9A and 7G. FACS analysis with CD44/CD24 markers was also performed immediately after sorting to control for the purity of the two populations CD44high/CD24low and CD44low/CD24high.
Gating strategy	To remove debris, dead cells and cell doublets SSC-A/SSC-H gating; cells with correct morphology were selected through FSC-A/SSC-A for both ECAD and CD44/CD44 FACS analysis.<br>For ECAD FACS analysis subsequent gating was based on cells stained with only secondary antibody (see Methods for details)<br>For CD44/CD24 double stainings subsequent gating was based on not stained and single stained populations.

☐ Tick this box to confirm that a figure exemplifying the gating strategy is provided in the Supplementary Information.
